# What Should Conceptual Engineering Be All About?

**DOI:** 10.1007/s11406-021-00367-x

**Published:** 2021-04-27

**Authors:** Manuel Gustavo Isaac

**Affiliations:** 1 Swiss National Science Foundation, Wildhainweg 3, 3001 Bern, Switzerland; 2 ARCHÉ Philosophical Research Centre, University of St Andrews, 17-19 College Street, St Andrews, Fife KY16 9AL Scotland

**Keywords:** Metaphilosophy, Philosophical methodology, Conceptual engineering, Concepts

## Abstract

Conceptual engineering is commonly characterized as the method for assessing and improving our representational devices. Little has been said, however, on how best to construe these representational devices—in other words, on what conceptual engineering should be all about. This paper tackles this problem with a basic strategy: First, by presenting a taxonomy of the different possible subject matters for conceptual engineering; then, by comparatively assessing them and selecting the most conducive one with a view to making conceptual engineering an actionable method, that is, a method that can be applied effectively and consistently to specific case studies. The outcome is that conceptual engineering should be all about concepts on pain of pragmatic inconsistencies otherwise.

## Introduction

Conceptual engineering is a burgeoning research field in analytic philosophy that focuses on how to assess and improve our representational devices. One of its key features is its normative agenda: Instead of describing the representational devices we already have and use, conceptual engineers typically aim to prescribe those we ought to have and use in order to ameliorate our performance at certain tasks (preventing fallacies, promoting group agency, formulating generalizations of explanatory value, etc.). Yet, still little has been said so far on how best to construe the representational devices that form the very subject matter of conceptual engineering.^1^ Typically:

Many of those who write about conceptual engineering are unclear on the exact nature of the entities being engineered. [...] That’s unfortunate because it makes the[ir] view[s] hard to assess—you don’t really have an account of conceptual engineering unless you make an explicit choice here. (Cappelen 2018:141)

This paper tackles this foundational problem head-on, namely: What should conceptual engineering be all about? In particular, it inquires whether or not conceptual engineering should be about concepts and why.^2^ To do so, my strategy is pretty simple: I start by presenting a taxonomy of the different possible subject matters for conceptual engineering; then I comparatively assess them and select the most conducive one with a view to making conceptual engineering an actionable method, that is, a method capable of being effectively and consistently applied to specific case studies. Throughout, we will deal with the label ‘conceptual engineering,’ what that label should mean (that is, its correlated concept), and the extension of that label’s meaning. Yet, before we tackle the problem of conceptual engineering’s subject matter, a couple of preliminary remarks seems appropriate.

## Against Anti-foundationalism

First, one may wonder whether and why foundational issues, such as that which concerns the subject matter of a method, really matter in the case of conceptual engineering. As it happens, one may be good at doing something without having any explicit understanding, or with only a fairly limited, implicit understanding, of what one is operating on, so that a practice would not need to rest on the foundational theorization of its subject matter in order to be well executed. Call this line of reasoning ‘anti-foundationalism.’

The problem with anti-foundationalism with respect to the subject matter of conceptual engineering is that conceptual engineering, from the standpoint of its application, is not a mere practice but a method. A mere practice can be characterized, for our present purposes, as the straightforward exercise of an activity. While it can thus be carried out, to a certain degree, in the absence of any reflective understanding (*a fortiori* theoretical) of the target objects it is operating on, a method cannot—lest it become utterly inefficient. The reason is that a method, as a way of doing something to attain a specific object (*meta-odos*), basically consists of a collection of principles and/or procedures, which themselves serve a given field of study.^3^ The field of study in question supplies these principles and/or procedures with the target objects they are operating on. Therefore, a method that does build on a theorization of its subject matter—be it made up of one or several different types of target objects (cf. Section 9)—is always more likely to be efficiently and consistently actionable (cf. Section 5).

In addition to that theoretical/methodological concern, one may add a matter of principle: The problem of conceptual engineering’s subject matter targets the very representational device ‘conceptual engineering’ (namely, its ‘conceptual’ component). Accordingly, addressing it should be top priority for conceptual engineers—for they are those supposedly in charge of criticizing any of our representational devices (cf. Sections 7 and 11).

## A Top-Down Approach

Second, once one acknowledges the critical importance of the problem of conceptual engineering’s subject matter, one should further distinguish between two symmetrical approaches to that foundational issue, each targeting different phenomena. On the one hand, a *top-down approach*, which assumes that the theorization of the subject matter of conceptual engineering ought to precede its application, as a method, to specific case studies, and which then stipulates what this subject matter ought to be, offering arguments for this stipulation. On the other hand, a *bottom-up approach*, which expects that a theory of conceptual engineering, including its subject matter, will eventually come up as a result of its application, as a method, to many specific case studies, and which thus aims to generalize on the many instances of engineering projects, trying to single out their common denominator (cf. Cappelen et al.n.d.). And one should then understand that addressing the subject matter problem properly requires adopting a top-down approach toward conceptual engineering.

Actually, neither of these two approaches is exclusive of the other. Both are in fact compatible and even complementary. But only one of them, the so-called ‘top-down’ approach, may serve to purposefully address the foundational issue that concerns the subject matter of conceptual engineering. Whereas, by contrast, its symmetrical counterpart, the so-called ‘bottom-up’ approach, may well bring some foundational insights, but only in some kind of erratic and indirect way, and it would thus sooner or later need be supplanted by a top-down perspective.

The reason is as follows: If one were to adopt a non-foundationalist approach to methods in general, building conceptual engineering on some piecemeal collection of resembling practices would call, at a certain point, for regimenting that collection in order to turn conceptual engineering into an efficiently and consistently actionable method—no legitimate need for any fancy new label otherwise. And for this purpose precisely, establishing top-down what conceptual engineering should be all about would always be required in the end. Therefore, the top-down approach is much more efficient strategy to begin with when it comes to making conceptual engineering an actionable method, that is, a method capable of being effectively and consistently applied to specific case studies.

That’s it for the preliminary remarks. Let’s now tackle the problem of conceptual engineering’s subject matter head-on.

## Taxonomizing the Subject Matter

Several different positions have been taken so far *vis-à-vis* the subject matter of conceptual engineering. From a top-down perspective, these can be taxonomized via a twofold alternative made of two successive dichotomies. The first alternative is a general one that consists of two opposite options: On the one hand, the *principled views* , which take conceptual engineering to be about some specified representational devices (viz., concepts, linguistic meanings, lexical items, conceptions, speaker-meanings, etc.); and on the other hand, the *unprincipled views* which, building on anti-foundationalism (be it implicitly or not), take conceptual engineering to be about any kind of representational device more or less indistinctly, that is, with no need for further specification (e.g., Burgess 2020; Burgess and Plunkett 2020; Cantalamessa 2019; Nado 2019; Prinzing 2018; Sterken 2020; Tanswell^2018^).

The second alternative is a specific one that distinguishes, within the principled views, between the two following opposite options: A *positive wing*, on which the representational devices that conceptual engineering is about are to be specified as concepts, sometimes conflated with linguistic meanings (e.g., Haslanger 2020; Isaac^2020^; Koch 2020; Machery 2017; Richard 2020; Scharp 2013, 2020); and a *negative wing*, on which these representational devices are not to be specified as concepts, but instead, for instance, as linguistic meanings, intensions, conceptions, or speaker-meanings (e.g., Cappelen 2018; Pinder 2019; Sawyer 2018, 2020a, 2020c).

This basic taxonomy roughly maps the landscape of positions *vis-à-vis* the subject matter of conceptual engineering.^4^ Only a minority of these views builds on strong theoretical justifications (namely, Cappelen 2018; Koch 2020; Machery 2017; Sawyer 2018, 2020a, 2020b, 2020c; Scharp 2013), while the absence of principled views clearly appears to be the dominant situation for projects in conceptual engineering. Now, I contend that, among these three available options (that is, the unprincipled views along with the positive and the negative principled views), only one is viable with a view to making conceptual engineering an actionable method—namely, the pro-concept one. In what follows, I provide a two-step argument as to why the alternative takes on conceptual engineering, by contrast, run the risk of making it a non-starter.

## The Actionability Principle

The first step of the argument to establish that the representational devices that conceptual engineering is about should be construed as concepts concerns the general dichotomy between the principled and the unprincipled views on the subject matter of conceptual engineering. More specifically, it consists in establishing that the representational devices that conceptual engineering is about should be specified, as defended by the principled views in opposition to their unprincipled counterparts— the alternative between the pro-concept and the no-concept wings being not at stake yet. As the unprincipled views build on anti-foundationalism about the subject matter of conceptual engineering, the argument to this effect goes along the same lines as Section 2 and draws on common sense.

In essence, conceptual engineering is first and foremost intended to be an actionable method.^5^ More precisely, a method that is both efficiently and consistently applicable, as concerns its procedures and principles, respectively, for processing its target objects in order to obtain the expected outcome (roughly, ameliorating our representation-involving cognitive activities). And it is only when a method builds on a theoretical understanding of its target objects—in light of the *OED*’s words (note 3), let’s assume this to be common sense—that it is the more likely to meet its actionability requirements (especially, when these are cashed out in terms of efficient and consistent applicability). In short: In order to be most actionable, a method ought to build on a theorization of its subject matter—call this ‘the actionability principle.’^6^ Therefore, with a view to making conceptual engineering an actionable method, one should start by “mak[ing] an explicit choice here” (Cappelen 2018: 141) and specifically identify its very subject matter.

Otherwise, indeed, if one were to disregard the need to ‘make an explicit choice’ on the subject matter of conceptual engineering, as the unprincipled views do, one wouldn’t “really have an account of conceptual engineering” (Cappelen 2018: 141). Rather, one would then most certainly relegate conceptual engineering to remaining some useless, piecemeal collection of resembling practices, as it would then intrinsically lack any overall grip over its target domain. The underspecification of the subject matter of conceptual engineering would thereby undermine one of the very core purposes of conceptual engineers, which is presumably to turn conceptual engineering into an actionable method. In other words, the unprincipled views on the subject matter of conceptual engineering deliver a pragmatically inconsistent (that is, counter-productive) option—at least, to those who are committed to making conceptual engineering an actionable method.

## The Self-Discrediting Predicament (I)

Once granted that the representational devices that conceptual engineering is about should be specified for the sake of making conceptual engineering an actionable method, the second step of the argument to establish that these should be construed as concepts concerns the specific dichotomy that distinguishes, within the principled views on the subject matter of conceptual engineering, between the ‘pro-concept’ and the “‘no concept’ wing[s]” (Scharp 2020: 412). More specifically, it consists in establishing that the specific representational devices that conceptual engineering is about should be identified as concepts, in opposition to any other kind of representational devices. The argument to this effect is pretty straightforward, as it proceeds from a *reductio*, which goes as follows:

If conceptual engineering is not (to be) about concepts, then ‘conceptual engineering’ is a misnomer.^7^ But if ‘conceptual engineering’ is a misnomer, then it is misleading. And if it is misleading, then it constitutes a cognitive impediment—typically, it will trigger wrong associations in people’s mind (just ask your undergrad students!). Therefore, the very label of conceptual engineering would contravene another of the very core purposes of conceptual engineers, that is: Creating better representational devices to foster better thinking, talking, and reasoning. It would then confront conceptual engineers with a self-discrediting predicament—some sort of ‘Do as I say not as I do’ syndrome.

## The Self-Discrediting Predicament (II)

Furthermore, as a corollary of its being a cognitive impediment, the label ‘conceptual engineering’ would be a deficient representational device, in crucial need of re-engineering. Being such a deficient representational device, it would then be an exemplary bad case of conceptual engineering. As Cappelen (2018: 53 <ms.>) concedes, on the no-concept wing, “‘conceptual engineering’ isn’t a great label (<or even a very bad one> [...]).” Now, given that such a very bad case of conceptual engineering would have been intentionally created by those who are supposed to enable us creating better representational devices, the no-concept wing of conceptual engineers would face here another self-discrediting predicament—this time, a version of the ‘Poorly shod shoemaker’ syndrome.^8^

Here one may think of calling for some kind of tolerance with regard to ‘conceptual engineering’ being a deficient representational device. But this would contravene yet another important principle of conceptual engineering—namely, the ‘representational skepticism’ that is meant to characterize the “intellectual attitude” underlying the very activity of conceptual engineering Cappelen(2018: 5–7, 2020). On such skepticism, indeed, conceptual engineers should never “uncritically take over the representational devices handed to them” (Cappelen 2018: 5). Consequently, being complacent to ‘conceptual engineering’ *qua* misnomer would amount to claiming for its immunity to conceptual engineering’s skeptic mindset—a sort of blind spot at the very heart of the whole enterprise. And granting oneself such privileges would arguably be reason enough to mistrust the legitimacy of conceptual engineers in the first place.

## Against Foundational Neutralism

Against my case for the specific identification of the subject matter of conceptual engineering as concepts, one could resist with an ecumenical take on our representational devices, as already alluded to as a third possible option among the principled views on the subject matter of conceptual engineering (see note 4). In particular, one could deliberately choose to either underspecify or overspecify the subject matter of conceptual engineering. Call this stance ‘foundational neutralism,’ as illustrated below by Burgess and Plunkett (2013: 1095):^9^

We would like to cast as wide a net as possible. Eliminativists about concepts will hopefully be able to massage our discussion to fit the mold of their favorite metaphysics of mental representation. [...] Theorists with diverse views on the nature of content ought to be able to engage in conceptual [engineering] without talking past each other.

Importantly, foundational neutralism differs from the anti-foundationalism that underlies the unprincipled views on the subject matter of conceptual engineering (cf. Sections 2 and 4). Anti-foundationalism straightforwardly dismisses the need to specify in whatever way the subject matter of conceptual engineering and thus leads to unprincipled views in this regard. By contrast, foundational neutralism is itself a principled view and as such it addresses the problem of conceptual engineering’s subject matter. It simply comes down to advising not to make an exclusive choice between the different representational devices that are eligible as target objects for engineering projects (e.g., concepts, linguistic meanings, lexical items, etc.), but instead to make the deliberate choice of including them all, so as to ‘to cast as wide a net as possible’ (Burgess and Plunkett 2013: 1095), be it by under- or by overspecification of the subject matter of conceptual engineering.

In case foundational neutralism were to build on the underspecification of the subject matter of conceptual engineering, being as minimal as it could be with respect to the characterization of the representational devices to be targeted by projects in conceptual engineering, it would then share the same shortcoming as anti-foundationalism: None would secure a proper theoretical grip for conceptual engineering over its target domain, be it on purpose or by carelessness; and thereby both would hinder alike the prospect of making it an efficiently applicable method (Section 5). On the other hand, in case foundational neutralism were to build on the overspecification of the subject matter of conceptual engineering, in the sense of intending to encompass the theorizations of each and every single type of representational device that is eligible as a target object for projects in conceptual engineering (viz., concepts, linguistic meanings, lexical items, speaker-meanings, etc.), it would then most certainly include, among the components of its method, principles that conflict as to the processing of its many different target objects. And thereby, it would hinder to the same extent the prospect of making it a consistently applicable method (ibid.). On both options, then, foundational neutralism would undermine alike the actionability prospect for the method of conceptual engineering. Hence, it should be discarded.

## The Pluralization of Engineering Projects

In light of the self-discrediting predicaments it faces, along with the shortcomings of foundational neutralism, ‘conceptual engineering without concepts [only]’ (Greenough 2020: 205) turns out to be a pragmatically inconsistent option all the way down: It leads to contradictions in the resulting practice of conceptual engineering as an applicable method. I consider such foundational, pragmatic inconsistencies to be sufficient grounds for simply dismissing it altogether. And as a consequence, I contend, we should stand with the positive wing and claim that conceptual engineering *should be* all about concepts—whatever it may have been (taken to be) so far.^10^

However, the pragmatic inconsistencies of foundational neutralism, the no-concept wing, and the unprincipled views, together with their resulting rejection as viable options for making conceptual engineering an efficiently and consistently actionable method, do not preclude the possible pluralization of engineering projects. In more concrete terms, the recommendation is here for engineers to put their own houses in order first—and thereby avoid the ‘blind spot issue’ that casts doubt on the legitimacy of their endeavor (Section 7). Basically, to contrast with the ecumenism of foundational neutralism, my advice is twofold. On the one hand, to re-label the various instances of engineering projects ‘semantic,’ ‘lexical’ or ‘terminological engineering,’ etc. in accordance with what alternative representational devices their subject matter is taken to be (namely, linguistic meanings, lexical items or terms, etc.).^11^ And on the other hand, to think about these different engineering projects accordingly, that is, each with its own appropriate concept (namely, SEMANTIC ENGINEERING, LEXICAL ENGINEERING, etc.).^12^ All being equally legitimate but distinct projects, pertaining to the general category of ‘representational devices engineering’ (Cappelen and Plunkett 2020: 3), and all possibly collaborating with one another on specific case studies. Importantly, pluralism thus construed is not an option for conceptual engineering, but rather for engineering projects in general, which then get to be split into different kinds depending on their target objects; otherwise, it conflates with foundational neutralism of the over-specifying kind (Section 8).

## The Merry Chaos Objection

A final tentative objection to the exclusive endorsement of the pro-concept wing in conceptual engineering and the correlated pluralization of engineering projects might be that ‘conceptual engineering’ is a name not a description, which as such simply refers to the activity that self-described conceptual engineers take themselves to be engaged in (Cappelen 2018: 3, 4) and which, moreover, happily works as an umbrella term, despite its mislabeling most of the cases it applies to. Along these lines, Cappelen and Plunkett (2020: 3) are clear on their branding strategy:

Why call it ‘conceptual’ engineering when it’s about representational devices more generally? Purely for aesthetic reason: ‘representational devices engineering’ doesn’t roll off the tongue in the way ‘conceptual engineering’ does.

As it happens, indeed, ‘conceptual engineering’ is a fancy label that has attracted both funding and people’s attention, and which immediately rings a bell to anyone within or outside philosophy as soon as they hear of it—at any rate, much more than ‘semantic,’ ‘lexical,’ ‘terminological engineering,’ and the like would. So, why not be a bit less hardline on foundational matters and let them call ‘conceptual engineering’ what, in principle, should be labeled otherwise? Call this move ‘the merry chaos objection.’^13^

In response to the merry chaos objection, I would start by acknowledging that, as a promotional strategy and when the target audience is that of professional philosophers (see note 17), keeping conceptual engineering in a messy state might be beneficial—you are of course likely to get more people on board this way than by saying that conceptual engineering has to be about concepts as partitions of logical space (Haslanger 2020), default bodies of information (Machery 2017), or interpretive common ground (Richard 2020), and so on. Yet I would then immediately add the qualifications explained in the next section, which I take to definitely undercut the viability of any no-concept only option with a view to making conceptual engineering an efficiently and consistently actionable method.

## The Decipherability Rebuttal

*Pace* Cappelen and Plunkett (2020: 3), the label ‘conceptual engineering’ has certainly not been dubbed so ‘purely for aesthetic reason,’ but instead, because of its ‘lexical effect’ (Cappelen 2018: 108, 122–134). Aesthetic reasons, as called on above, would be about the human phonatory apparatus and the meaningless phonetic string , so that it ‘roll[s] off the tongue’ well; or about our visual system and the corresponding meaningless graphemic string, so that it pleases our eyes. By contrast, as I understand them, lexical effects are these cognitive and non-cognitive effects that result from the connection a signifier has to what it signifies.^14^ And this connection, although radically arbitrary at the morphematic level, is ‘motivated’ (as some linguists say) at higher syntagmatic levels, once items are embedded in linguistic systems.^15^ Typically, in the case of a compound noun phrase such as ‘conceptual engineering,’ the preposed adjective modifies the noun by specifying what it is about. Some compound noun phrases are not such, granted. But most of these were metaphors that became ‘demotivated’ (as some linguists say). Whereas ‘conceptual’ in ‘conceptual engineering’ was not coined as a metaphor, or so I take it—and the label is too recent anyway to have gone through a demotivation process. In short, you cannot mean anything by any wording, on pain of being misleading otherwise.

The upshot is then a *Golden Rule for lexical engineering*: When the coining of a new technical term builds on extant linguistic items that are themselves already meaningful (viz., morphemes or lexemes), the new technical term should obey decipherability (or transparency) requirements. In other words, it should enable the receivers to correctly infer the intended information from the signifier itself (viz., some phonetic or graphemic string). Even more so when its composing items also pertain to non-technical languages and when it is furthermore meant to be used in non-expert communities. Otherwise, they are misleading and harmful. As when non-literal uses of some lexical items are presented as being literal—such as ‘conceptual engineering’ when used to mean NON-CONCEPTUAL ENGINEERING or NON CONCEPTUAL-ONLY ENGINEERING, and to refer to the corresponding activities.^16^ The simple reason is that, in virtue of them having been intentionally created, new technical terms, when made of already meaningful linguistic components, are always expected to be motivated, and never radically arbitrary.

In light of so-called ‘lexical effects’ and the correlated decipherability requirements, conceptual engineers without concepts (only) thus faces the following dilemma, neither of whose horns seems acceptable. Either to acknowledge that ‘conceptual engineering’ is a defective representational device, in crucial need of re-engineering, no matter whatever metalinguistic caveat (cf. note 16). But then, they will still be confronted by a self-discrediting predicament.^17^ Or to acknowledge that they are ‘exploiters’ (Cappelen 2018: 133–134), in the branding business of trying to label their views so that they trigger to desired kinds of (non-)cognitive effects, no matter how misleading.^18^ Bottom-line: The merry-chaos option would then make ‘conceptual engineering’ a bad label for a bad idea, as some would happily have it—and so I claim.

## Conclusion

This paper has been about what conceptual engineering should be all about. The main problem it has addressed is that of the subject matter of conceptual engineering, namely: How best to construe the representational devices that form the target domain of engineering projects? This problem in turn has been subdivided into two successive issues: Firstly, should the subject matter of conceptual engineering be explicitly specified, instead of remaining underspecified? If so, secondly, should the subject matter of conceptual engineering be explicitly specified as made of concepts, instead of any other kind of representational apparatuses (e.g., lexical items, linguistic meanings, intensions, etc.)? By tackling these foundational issues stepwise, I came to successively narrow down the subject matter of conceptual engineering in the claim that, with a view to making it an actionable method, conceptual engineering should be all about concepts—namely, on pain of being pragmatically inconsistent otherwise. A last resort worry might be that all this is but of little contribution to ongoing debates in conceptual engineering. Fortunately, this worry is very easy to dissipate. Just by recalling a few salient facts.

First, mainstream conceptual engineering does not care about specifying in any way its target domain. Second, within the minority of those who have deemed it worthwhile to specify their target objects, the most dominant source has advocated for an anti-concept version of conceptual engineering (Cappelen 2018). Third, none of the remaining few who have endorsed a pro-concept version of conceptual engineering have ever provided any argument as to whether and why conceptual engineering should be about concepts. Fourth and finally, skepticism towards concepts is thus widespread among conceptual engineers. As a consequence, however simple the claim may be, the argumentative justification for conceptual engineering to be about concepts, not only is novel, original, and needed, but it might as well dramatically reorient the investigations into the foundations of conceptual engineering—while opening, at the very same time, new prospects for alternative engineering projects (semantic, lexical, terminological, etc.) and their combination.

Arguably, ‘concept’ may mean many different things, so that the next crucial step in analyzing into the theoretical foundations of conceptual engineering is then to come up with an account of how to best conceive concepts for the purposes of conceptual engineering. In fact, Cappelen (2018: 141) has already put the challenge forward:

The first item on the agenda for such views [viz., those which take conceptual engineering to be about concepts] should be to specify what concepts are, and then present an account of how concepts so construed can be engineered.

Let’s be earnest then, and take it up. On the next occasion.

## Footnotes

1 Exceptions are Cappelen (2018), Koch (2020), Machery (2017), Sawyer (2018, 2020a, 2020b, 2020c), and Scharp (2013).

2 As we will see, this leaves open the possibility for the subject matter of engineering projects in general—and not projects in conceptual engineering specifically—to be made up of different types of target objects (Section 9).

3 See ‘method’ in the *OED*: “The principles or procedures of any mode or field of cognitive activity, themselves considered as an object or branch of study” (entry I.2d); “A special form of procedure or characteristic set of procedures employed (more or less systematically) in an intellectual discipline or field of study as a mode of investigation and inquiry” (entry I.3a).

4 For the sake of clarity, a third option within the principled views is left aside until Section 8, namely, that which advocates for the neutrality of conceptual engineering *vis-à-vis* its subject matter by explicitly choosing to include any kind of representational device in it.

5 Cappelen’s (2018: 72–74) ‘lack of control-will keep trying’ principle appears to be the exception here, as it somehow turns conceptual engineering into a worthwhile enterprise in spite of it being doomed to remain infeasible.

6 By way of analogy, one could think, for instance, of methods to learn foreign languages: With no theoretical understanding of their target language (in terms of phonetics, morphology, syntax, or whatever), these would all be useless.

7 Cf. Cappelen (2018: 104): “Conceptual engineering and amelioration are mislabeled: it’s not about improving concepts—in fact, it’s not about concepts at all.”

8 One might feel that the self-discrediting predicament, on both its variants, is a bit off track as it targets the label ‘conceptual engineering’ instead of the concept of conceptual engineering. But this would be misguided, for the argument has to be run at a neutral level on which one only knows that the subject matter of conceptual engineering has to be specified, without knowing as what kind of representational device—on pain of committing a *petitio principi* otherwise. That is, at a level on which what one practices in fact is ‘representational devices engineering.’

9 Relatedly, Burgess and Plunkett (2013: 1094) consider the option of re-labeling their enterprise ‘ethics of representation,’ but eventually come to reject it on the ground that “the R-word seems to carry strong inflationary connotations” with respect to truth and reference, whereas the things that conceptual engineering is about (whatever they are) may well not “intrinsically or essentially [be] representational in an inflationary sense”.

10 One might worry that such a prescriptive take on the subject matter of conceptual engineering runs the risk of causing some discontinuity of inquiry in conceptual engineering, so much so that its future implementation would then be disconnected from its past and ongoing practice. But, first, defending the pro-concept wing amounts to reducing the practice of conceptual engineering to one of its extant variants (Section 4). And, second, if this were to cause some discontinuity, this would be but rightly so, as the topic would have needed to be changed anyway.

11 Just to be clear: This means that the name has to square with the target practice, not the other way around. So that, if the activity of conceptual engineers would have ended up being labeled ‘car engineering,’ then it should be re-labeled—and conversely if ‘conceptual engineering’ were to label the activity of car engineers. As we will see below, what is at stake here is a matter of decipherability (Section 11).

12 Note that, in this light, my disagreement with those who think of conceptual engineering and use its label as being not about concepts, but about some other representational device instead (Section 4), is not merely verbal. Thanks to an anonymous reviewer (this journal) for pressing me on this point.

13 Note that the merry chaos objection may result from anti-foundationalism (Section 2), just as well as it can be theoretically motivated by foundational neutralism on the subject matter of conceptual engineering (Section 8).

14 Cappelen seems to conceive of lexical effects as being grounded on meaningless strings of symbols, independently of their semantic properties (e.g., Cappelen 2018: 123, 131), in which case, they shouldn’t have been called ‘lexical,’ since lexical units are meaningful per definition (namely, as the pairing of a sense with a lexical form).

15 Take ‘concept,’ for instance, with a bit of folk etymology in mind: Of course, Latin *con-* and Proto-Indo-European **kap-* arbitrarily signify ‘together, with’ and ‘to grasp,’ respectively; but when they combine, via *capere* (‘to take’), to form *concipere* (‘to take in and hold’), which led to *conceptum* via past-participle stem *concep-*, they obviously cannot signify, e.g., what *praecipere* signifies (namely, ‘to give rules to, order, advise’).

16 In the case of conceptual engineering being not about concepts only, careful use of the misnomer could presumably include a warning for it being taken in a metonymic way, such as ‘in a broad sense’ or something in this vein. But then, it will either block and loose the effect it expects to trigger or the warning will have been added as a form of disclaimer, in the hope that it will go unnoticed, while the label itself will thus remain misleading.

17 The potential target user audience will also most certainly shrink to the insider community of fellow professional philosophers: Who else indeed would be willing to buy the idea that conceptual engineering is not about concepts? Try to apply to some major funding body with it (or ask your undergrads again) and you will see. I bet all will be baffled and rather doubt your ability to be up to the task you set up forth: Creating better representational devices!

18 As Cappelen (2018: 129/130) puts it: “It might turn out that even for us, a significant amount of our work consists in trying to trigger the right kinds of lexical effects. [...] [M]ost likely a name can also make you more disposed to accept, say, a philosophical theory (it is, after all, cheaper to accept a philosophical theory than to buy a bottle of Coke, so we should expect the barriers to be lower).”
